# Piezo1: the key regulators in central nervous system diseases

**DOI:** 10.3389/fncel.2024.1441806

**Published:** 2024-10-30

**Authors:** Yi Xu, Yuheng Wang, Yanling Yang, Xiaowei Fang, Lidong Wu, Jialing Hu, Jin Li, Shuchong Mei

**Affiliations:** ^1^The Second Affiliated Hospital of Nanchang University, Jiangxi Medical College, Nanchang University, Nanchang, China; ^2^Department of Emergency Medicine, The Second Affiliated Hospital of Nanchang University, Jiangxi Medical College, Nanchang University, Nanchang, China

**Keywords:** Piezo1, CNS diseases, biological properties, Ca2+, pharmacology

## Abstract

The occurrence and development of central nervous system (CNS) diseases is a multi-factor and multi-gene pathological process, and their diagnosis and treatment have always posed a serious challenge in the medical field. Therefore, exploring the relevant factors in the pathogenesis of CNS and improving the diagnosis and treatment rates has become an urgent problem. Piezo1 is a recently discovered mechanosensitive ion channel that opens in response to mechanical stimuli. A number of previous studies have shown that the Piezo channel family plays a crucial role in CNS physiology and pathology, especially in diseases related to CNS development and mechanical stimulation. This article comprehensively describes the biological properties of Piezo1, focuses on the potential association between Piezo1 and CNS disorders, and explores the pharmacological roles of Piezo1 agonists and inhibitors in treating CNS disorders.

## 1 Introduction

Diseases of the central nervous system, such as gliomas and strokes, and neurodegenerative diseases, such as Alzheimer’s disease (AD) and Parkinson’s disease (PD), affect millions of people worldwide. The prevalence of neurodegenerative diseases has increased in the last few decades owing to an aging global population and has reached epidemic proportions (Brayne and Miller, 2017). However, little is known about its pathogenesis. According to recent estimates, as of 2019, approximately 5.8 million Americans will be diagnosed with AD, with a prevalence rate predicted to be 3–7% and increasing with age. Age is also a major imaging factor, with men being about 19–29% less likely to develop AD than women (Khan et al., 2020; Soria Lopez et al., 2019). The main pathological alteration in AD is the accumulation of amyloid and dysfunctional tau proteins in the brain (Pluta et al., 2020). The prevalence of PD, a common neurodegenerative disorder, is approximately 2% (Polymeropoulos et al., 1997). It is characterized by the loss of dopaminergic neurons and formation of cytoplasmic protein inclusion bodies.

Piezo channels are a class of mechanosensory-sensitive membrane proteins whose structural integrity and functional stability are critical for normal physiological activity in living cells, and one of the most common classes is the Piezo1 ion channel (Zhou and Martinac, 2023). Piezo1 is a mechanosensitive cation channel with broad physiological significance that plays a key role in the CNS (Zheng et al., 2023). Mechanotransduction refers to the fundamental ability of organisms to receive and also respond to physical signals from internal and external environments and is found and characterized in vertebrates, invertebrates, plants, and protozoa. Abnormal or faulty mechanotransduction may lead to a range of diseases, such as deafness (Lee et al., 2023), cardiovascular disease (Lyon et al., 2015), metabolic defects (Romani et al., 2021), fibrosis (Duscher et al., 2014), cancer metastasis (Broders-Bondon et al., 2018), neurological disorders (Ostrow and Sachs, 2005), and osteoporosis (Burger and Klein-Nulend, 1998).

Mechanical signal transduction involves the direct stimulation of mechanically activated (MA) ion channels by mechanical forces applied to the cell membrane, which convert mechanical forces into biochemical signals and thus respond rapidly and efficiently to the excitation of the cell membrane or the activation of intracellular signals (Qin et al., 2021). In the last decade, considerable research has been conducted on the biomechanical properties of the CNS in both health and disease. These past studies indicated that not only chemical but also mechanical cues could drive the progression of many pathophysiological states (Velasco-Estevez et al., 2022). Piezo1 is the primary MA channel. Activation of Piezo1 channels by mechanical stimulation leads to Na+/Ca2+ inward flow, which triggers electrical signals and initiates a second messenger signaling pathway (Harraz et al., 2022). In this paper, we have provided a comprehensive description of the structure and function of Piezo1, a discussion of the pathophysiological role of Piezo1 channels in CNS diseases, and an examination of the pharmacological properties of Piezo1 channel antagonists or inhibitors as targeted therapies to reduce its expression in CNS diseases.

### 1.1 A summary of Piezo1

In 2010, Patapoutian et al. discovered a new group of mammalian MA ion channels and designated this group as the Piezo family (Arnadottir and Chalfie, 2010). This groundbreaking research, for which the Patapoutian was awarded the 2021 Nobel Prize in Physiology or Medicine, attracted much attention from many researchers worldwide and successively reported the functions of two members of the Piezo1 and Piezo2 families in different tissues and organs. Over the past 10 years, studies using experimental mouse models have shown that Piezo1 was predominantly expressed (Arnadottir and Chalfie, 2010) in non-excitable cell types, such as vascular endothelial cells and erythrocytes (Jiang et al., 2021), in contrast to Piezo2, which was predominantly expressed in sensory neurons. Accumulating evidence suggests that Piezo1 is required for vascular development and function (Retailleau et al., 2015), erythrocyte volume regulation (Cahalan et al., 2015), intraepithelial homeostasis (Eisenhoffer et al., 2012), neural stem cell lineage selection (Pathak et al., 2014), axonal growth (Koser et al., 2016) and urinary osmolality (Martins et al., 2016). Piezo2 plays an essential role in sensory processes such as tender touch, sensation (Maksimovic et al., 2014), response to mechanical injuries (Kim et al., 2012), proprioception (Woo et al., 2015), and hearing (an indispensable function) (Wu Z. et al., 2017). It also plays a role in gastrointestinal (Bai et al., 2017) and respiratory physiology (Wang et al., 2017). In this paper, we have focused on the role of Piezo1 in CNS diseases.

### 1.2 Pathophysiological role of Piezo1 in the CNS

Recent studies have shown that mechanical forces determined neural development and function (Abuwarda and Pathak, 2020). Mechanical signals encountered during development include changes in tissue stiffness, fluid shear flow, and hydrostatic forces in the cerebrospinal fluid from the developing ventricles, all of which can affect the expression of Piezo1 channels. MA ion channels can open rapidly in response to mechanical forces and modulate several downstream effects via electrochemical signaling. This typically includes mechanical anomalous pain, tenderness, and pressure receptor reflex.

A previous study (Pathak et al., 2014) showed that traction-mediated Piezo1 activation triggers inward calcium flow and directs neural stem cell lineage selection toward a neuronal phenotype than the astrocyte phenotype. Piezo1 is expressed on the neuronal cell membrane in the vertebrate CNS and regulates important developmental processes, such as axon guidance. This function was recently demonstrated during the development of retinal ganglion neurons in African Javanese embryos (Koser et al., 2016). Other studies have shown that Piezo1 channels are expressed in both neuronal and non-neuronal cell types in different brain regions (Velasco-Estevez et al., 2018; Wu J. et al., 2017). In addition, many Piezo1 channels are expressed on the surface of cerebral vasculature, cerebral aqueducts, and spinal cord central canal endothelial cells (Harraz et al., 2022). Capillary endothelial cells play an important role in neurovascular coupling, and Piezo1-mediated capillary mechanosensation may be involved in this process (Harraz et al., 2022). Recent reports have shown that the shear-induced opening of endothelial Piezo1 channels activates the calcium-dependent protease calpain (Li et al., 2014), which in turn cleaves talin (a protein that links membrane integrins to the actin cytoskeleton) to alter integrin-mediated cell adhesion (McHugh et al., 2010). Activated Piezo1 also recruits the small GTPase R-RAS to the endoplasmic reticulum, leading to the release of internally stored calcium. Past studies have shown that excessive activation of Piezo1 by the Piezo1 agonist Yoda-1 may lead to CNS axon demyelination via excessive calcium-mediated destabilization of integrin signaling (Harraz et al., 2022).

Recent studies have shown that feed-forward circuits mediated by Piezo1 and tumor histomechanics promote glioma growth in CNS (Chen et al., 2018). Increased intratumoral solid pressure during high-grade glioma formation provides mechanical signals that stimulate Piezo1, which plays an important role in tumor cell proliferation and metastasis. For example, in hepatocellular carcinoma (HCC), matrix stiffening is manifested at the cellular and tissue levels by increased activation and expression of the Piezo1 gene, which in turn promotes the secretion of substances such as VEGF and CXCL16, as well as increased levels of the ubiquitylation of HIF-1α, which ultimately manifests itself as a substantial increase in the rate and extent of vascular growth of HCC and promotes metastatic spreading of cancer cells (Li M. et al., 2022). Previous studies have shown that high levels of Piezo1 predict a poor prognosis (Zhou et al., 2020). The incidence and severity of CNS diseases such as AD, PD, and stroke were significantly positively correlated with the degree of piezo1 gene expression.

## 2 The structure and features of Piezo1

The main MA cation channels essential for mammalian cell physiology are Piezo1 and Piezo2. Human Piezo1 protein was originally identified in mice (Coste et al., 2010). Coste et al. tested approximately 75 genes and found that one gene, Fam38A, was important. The protein encoded by this gene is not similar to that of the Piezo1 channels. A homologous gene (known as piezo2/fam38b) from dorsal root ganalion (DRG) cells was cloned (Coste et al., 2012).

Piezo1 and Piezo2 are located on chromosomes 16 and 18, respectively. Human Piezo1 consists of 2,521 amino acids. Each is approximately 300 kDa in size and is likely glycosylated (Beech and Kalli, 2019).

Studies have shown that human (Schrenk-Siemens et al., 2015), zebrafish (Faucherre et al., 2013), chicken (Soattin et al., 2016), Drosophila (He et al., 2018) bird (Schneider et al., 2014), mouse (Ikeda et al., 2014; Maksimovic et al., 2014), meadowlark (Coste et al., 2010), and African clawed frog Piezo1s were homologous (Methfessel et al., 1986). Numerous studies showed that Piezo1 is expressed in most mammals, and that it is widely expressed in various organs and tissues in humans, including the brain, lungs, gastrointestinal system, bladder, and bones (Cahalan et al., 2015; Wu J. et al., 2017).

Functional mutations in human Piezo1 lead to the development of hereditary stem cell aplasia (also known as dehydocytosis) and familial anemia (Fang et al., 2021). Nonfunctional mutations lead to the development of extensive lymphoid dysplasia, which is characterized by varying degrees of anemia (Fotiou et al., 2015; Lukacs et al., 2015).

Recent studies have established the relevance of Piezo1 in vascular biology, with structural Piezo1 knockout (KO) leading to embryonic death within days due to apparent malformations in the vascular development in mice (Hyman et al., 2017; Li et al., 2014).

Piezo1 proteins are arranged in plain view to form the b-propeller domain of a trilobed protease, which allows the central ion pore to sense mechanical forces (Lim et al., 2018; Xiao, 2020; Zhao et al., 2018; Zheng et al., 2019). The Piezo1 extracellular propeller structural domain acts as a sensor of flow-related shear and other mechanical stresses (Hyman et al., 2017; Li et al., 2014).

Using cryo-electron microscopy, several features of mouse Piezo1 channel morphology were found to be identical to those of humans. In terms of structure and function, Zhao et al. In their study divided Piezo1 channels into three functional parts based on structure and function: (1) the ion-conducting pore part (the C-terminus); (2) the anchor, the C-terminal domain (CTD) and the beam, which are the conversion elements; and (3) the mechanosensing part composed of the transmembrane (TM) blades (Zhao et al., 2019). The channel structure has been described in detail in the literature (Fang et al., 2021; Xiao, 2020; Zhao et al., 2018).

Piezo1 channels have three interchangeable states of activity, i.e., closed, open, and inactivated (Gnanasambandam et al., 2015). The protein-lipid dome is attached to the membrane by a curved lipid bilayer region that produces a characteristic membrane footprint (Coste et al., 2012). Therefore, changes in the lipid bilayer composition directly affect membrane fluidity and alter the kinetics and function of Piezo1 channels (Ivkovic et al., 2022).

When mechanical stimuli affect the cell membrane, stress is distributed to all components, including the bilayer, cytoskeleton (CSK), and extracellular matrix (ECM), which converge on Piezo1 channels, inducing a shift from the closed to open state, a phenomenon that allows the plasma flow of calcium, potassium, and sodium ions (Ridone et al., 2019).

When Piezo1 proteins are subjected to mechanical forces, including shear stress, expansion, stretching, and compression, they release cations, such as calcium, potassium, and sodium ions, which promote cellular excitation and signaling (Li et al., 2014). Piezo1 channels act as shear stress or stretch sensors and play key roles in various mechanotransduction pathways, including embryonic vascular development (Li et al., 2014; Ranade et al., 2014), blood pressure regulation (Wang et al., 2016), hypertension-induced arterial remodeling (Retailleau et al., 2015), rapid epithelial cell division (Eisenhoffer et al., 2012; Maksimovic et al., 2014), stem cell differentiation (He et al., 2018; Pathak et al., 2014), sensory mechanical stimulation, causing bladder stretching (Martins et al., 2016), and stress-induced acute pancreatitis (Liu S. et al., 2021). The Piezo1 current is comparable to that of Piezo2 but has significant kinetic and conductance differences. Inactivation of Piezo1 is faster, and unit conductance and expression levels are lower than those of Piezo2 (Shinge et al., 2022). In mammals, Piezo1 channels are abundantly present in the mechanosensitive cells of the bladder, skin, lungs, kidneys, and colon.

Various structural and functional studies have revealed that Piezo1 channels possess multiple functions and these studies have demonstrated their association with other membrane proteins, such as platelet endothelial cell adhesion molecule (PECAM) (Beech and Kalli, 2019), ECM, focal adhesion zone (FAZ), adhesion junctions (AJs) and cytoskeletal structures (Nourse and Pathak, 2017). Nourse et al. provided a detailed review of the role of Piezo1 in mechanical sensing and interaction with the cytoskeleton (Jiang et al., 2021; Nourse and Pathak, 2017). One distinctive feature characterizing the overexpression of Piezo1 channels in cell lines is their rapid and complete inactivation (shutdown to a refractory state), which is often described as occurring within 50–100 ms after activation and is caused by an almost instantaneous pressure pulse (Coste et al., 2010; Wu J. et al., 2017). This feature has become a hallmark of these channels. Its structural domain has been identified (Zheng et al., 2019), and is likely a disease-inducing mechanism (Albuisson et al., 2013).

## 3 Relationship of Piezo1 to the CNS

As a class of cation-selective channels, Piezo1 responds to mechanical stimuli and is activated and rapidly inactivated in a voltage-dependent manner (Coste et al., 2010; Gottlieb et al., 2012; Moroni et al., 2018). This suggests that Piezo1 channels are associated with normal neuronal development, nociception, and pathological conditions (Fang et al., 2021; Gottlieb and Sachs, 2012). The following sections describe the role played by Piezo1 channels under normal and abnormal CNS conditions, which will help strengthen our understanding of Piezo1.

### 3.1 Effects of Piezo1 under normal conditions

Piezo1 regulates the number of neuroepithelial cells, and it is also involved in maintaining their integrity. The results of a previous study indicated that the neuroepithelium of Piezo1- KO embryos was thinner than that of their wild-type (WT) littermates, primarily because Piezo1- KO reduced the level of cholesterol synthesis in the CNS and the neutral lipid and cholesterol content in neural stem cells (NSCs) (Nourse et al., 2022). Piezo1 channels play a role in sensing mechanical properties in the microenvironment of neural progenitor cells, which allows the initiation of signaling pathways for neuronal differentiation and also determines the subsequent morphological structure of nerve fibers while altering the permeability of the glial membrane and activating its signaling pathways for nanosizing (Wu J. et al., 2017). The anterior cingulate cortex (ACC) plays a significant role in the transmission of nerve pain. Injury to the peripheral nervous system causes hyperactivity of ACC neurons and upregulation of Piezo1 expression, thus supporting the involvement of Piezo1 channels in pain information transmission. These channels may also be selectively expressed in the mouse DRG and are currently thought to primarily mediate nociception (Li Q. Y. et al., 2022; Roh et al., 2020; Wang et al., 2019). Piezo1 channels also mediate Ca2+ transients, and thus complete vascular pathfinding guided by endothelial tip cells to establish highly complex but delicate vascular networks in the brain that play an essential role in the mechanosensation of capillary blood flow in the CNS (Cudmore and Santana, 2022; Liu S. et al., 2020). Mechanosensing mediated by mechanosensitive channels such as Piezo1 is particularly important for axonal growth in the brain, and Piezo1 channels can be found to play a role in the negative regulation of axonal development, resulting in sensory and motor neurons that exhibit limited repair capacity (Koser et al., 2016; Song et al., 2019). For example, although Piezo1 promotes the growth and development of oligodendrocytes (OPCs) and oligodendrocyte progenitor cells in the human brain, its expression decreases with *in vitro* maturation of MO3.13 oligodendrocytes (Velasco-Estevez et al., 2022). We speculate that this decrease prevents the unbridled growth of neurons in the organism and facilitates a reduction in energy loss.

### 3.2 Effects of Piezo1 under abnormal conditions

Under normal conditions, Piezo1 expression plays an integral role in signaling in the central nervous system. The absence or overexpression of Piezo1 leads to abnormalities in the organism, such as hippocampal dysfunction (Xiong et al., 2022), cavernous cerebral malformation (Scimone et al., 2022), peritumoral brain edema (Qu et al., 2020a), and CNS demyelination (Velasco-Estevez et al., 2020). Previous studies have shown that mice with Piezo1 channels specifically knocked out in astrocytes exhibit severe hippocampal volume defects and a significant reduction in brain weight, which in turn leads to severe impairment of adult neurogenesis (Chi et al., 2022; Xiong et al., 2022). Further analysis revealed that the elimination of astrocytic Piezo1 significantly inhibited the proliferation of hippocampal NSCs and led to a significant reduction in the number of neural precursor cells, immature neurons, mature neurons, and astrocytes (Lee et al., 2021). The opposite effects were observed with upregulated expression of Piezo1. A typical example is neuroinflammation (Li Q. Y. et al., 2022). Certain inflammation-related factors, such as lipopolysaccharides, can activate Piezo1 channels, triggering both the influx of calcium ions and also the release of intracellular stores of calcium ions, leading to oxidative cellular damage and disintegration of the CNS cytoskeleton, manifesting as irreversible neurodegeneration (Arishe et al., 2020; Frati et al., 2017; Lee et al., 2021). Small injections of the Piezo1 agonist Yoda1 into the basal forebrain (BF) of mice produced the same pathological manifestations observed after 6 h of sleep deprivation, including long- or short-term fear memory deficits in mice (Ma et al., 2022). Mechanosensitive Piezo receptors (particularly the Piezo1 subtype) are found in the injury perception of the peripheral trigeminal nerve, and high-dose injections of Yoda1 can extend the meningeal arteries innervated by the trigeminal afferent nerve, leading to the development of migraines (Dolgorukova et al., 2021; Mikhailov et al., 2019). The expression of the Piezo1 gene and the production of the Piezo1 protein increase significantly with age, which explains the high prevalence of major neurological diseases in older individuals, such as the detection of Piezo1 expression in activated astrocytes surrounding amyloid plaques in patients with AD (Liu J. et al., 2021; Segel et al., 2019). Glioma molecular subtypes and clinical manifestations are highly compatible with Piezo1 expression, as glioma is one of the most prevalent primary malignancies in the adult CNS (Kaushik and Persson, 2018; Zhou et al., 2020). When Piezo1 is overexpressed, glioma cells exhibit a high degree of activity, leading to rapid proliferation and invasion. This suggests an association between the spread of cancer and glioma cells (Yu and Liao, 2021). Similarly, Piezo1 plays a negative regulatory role in myelin formation in the CNS, and when highly expressed, may lead to demyelination (Velasco-Estevez et al., 2020). Patients with diabetes often exhibit upregulation of Piezo1 expression, which causes microglial cell damage over time (Liu H. et al., 2021). Changes in mechanical forces may also increase the activation of Piezo1, which worsens ischemic brain damage (Guo et al., 2021). Therefore, we concluded that Piezo1 causes CNS damage under abnormal conditions.

## 4 Pathological expression of Piezo1 in CNS diseases

### 4.1 Pathological expression of Piezo1 in AD

Amyloid plaques are one of the main pathological features of AD and are formed by the accumulation of neurotoxic beta-amyloid protein (Aβ) in the brain (Ivkovic et al., 2022; Jantti et al., 2022; Liu H. et al., 2022; Velasco-Estevez et al., 2018). AD can be divided into two groups, late-onset AD (LOAD) and early-onset Alzheimer’s disease (EOAD). Most cases of AD are LOAD and are associated with incomplete clearance of Aβ (Mawuenyega et al., 2010; Yang et al., 2018). Early-onset AD is associated with increased Aβ production by neurons (Beutner et al., 2013; Hickman et al., 2008; Jantti et al., 2022). Therefore, targeted removal of Aβ is a potential therapeutic strategy in the treatment of AD.

The brain is softer and more sensitive than other tissues in the body, and its soft tissues are very sensitive to mechanical stimulation. Amyloid plaques can produce stiff mechanical stimulation of the brain tissue, which activates the immune function of astrocytes and microglia, leading to the engulfment of glial cells (Ivkovic et al., 2022; Jantti et al., 2022; Liu H. et al., 2022; Velasco-Estevez et al., 2022; Velasco-Estevez et al., 2018). Piezo1 is a member of the mechanically gated cation channel family, and its expression is higher in the CNS pathological state than in the normal physiological state (Velasco-Estevez et al., 2022).

**Figure 1:**
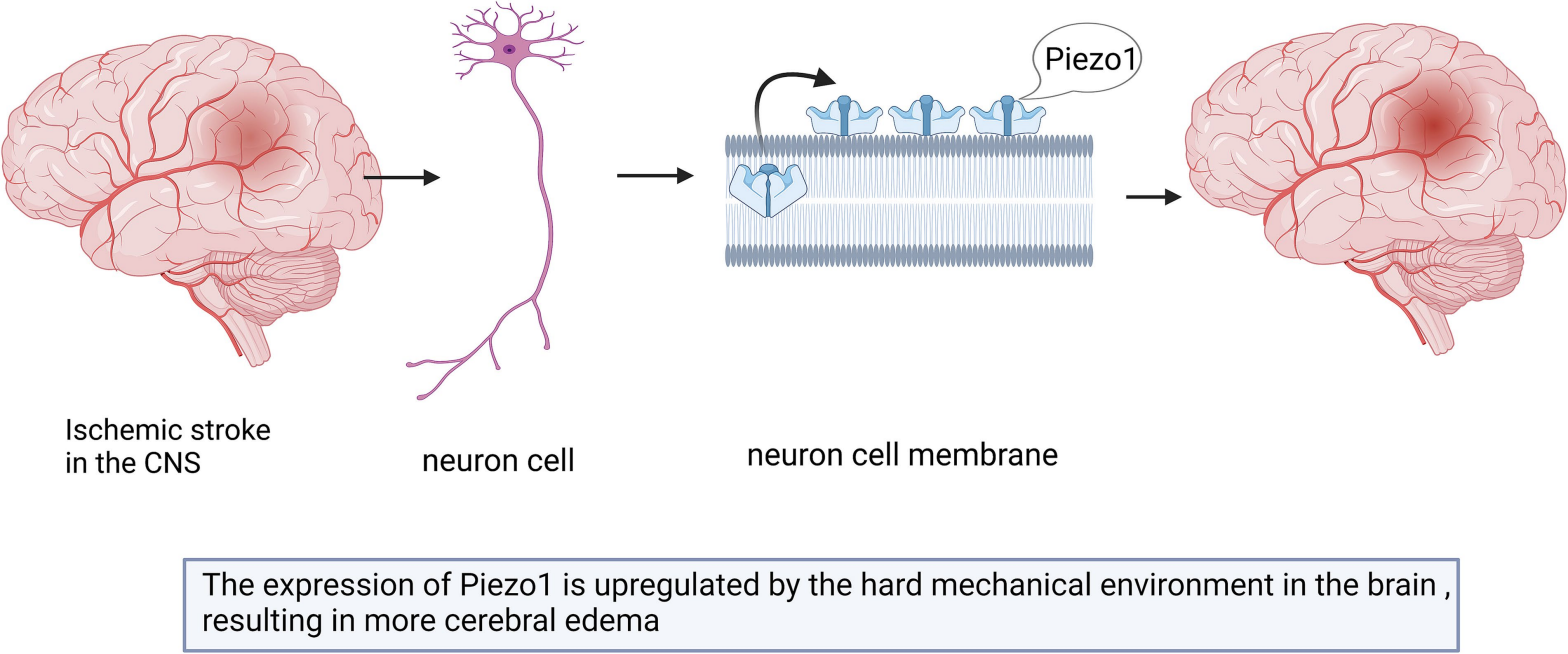
Schematic diagram of the action of Piezo1 on astrocytes and microglia in AD brain. The expression of Piezo1 is up-regulated in astrocytes, for unknown reasons, and astrocytes proliferate to phagocytose amyloid plaques. The stiff amyloid plaques may also upregulate Piezo1 channels in microglia cells, thereby releasing more inflammatory mediators to repair neurons.

Astrocytes can support and separate nerve cells and play a role in the formation of the blood–brain barrier (Beutner et al., 2013; Johnson et al., 2014). The loss of Piezo1 channels in astrocytes leads to a dramatic reduction in hippocampal volume and brain weight, thereby influencing adult neurogenesis and cognitive functions (Chi et al., 2022). As shown in Figure 1, astrocytes surrounding stiff amyloid plaques in the AD brain can upregulate Piezo1 channels (Liu H. et al., 2022; Velasco-Estevez et al., 2018). The underlying pathological mechanisms are unknown and need to be further investigated in depth in the future. However, microglial responses seem to overshadow the astrocytic contributions (Velasco-Estevez et al., 2018). The results of this study showed that Piezo1 in microglia plays an important role in AD pathology (Jantti et al., 2022).

Microglia are the resident immune cells of the CNS that have functions similar to those of astrocytes. The morphology of these cells depends on the local gradient in tissue stiffness (Jantti et al., 2022; Velasco-Estevez et al., 2018). As shown in Figure 1, microglia change their phenotype and release inflammatory mediators to repair damaged brain tissues (Ivkovic et al., 2022; Jantti et al., 2022).

Piezo1 can alter the phenotype and activity of microglia by sensing different mechanical stimuli in the environment (Ikiz et al., 2024). Macrophages, including microglia, tend to migrate toward regions of stiffer and denser mechanical gradients, a process known as tropism (Blaschke et al., 2020). Stiff amyloid plaques may upregulate Piezo1 channels in microglial cells and affect their immune activity; however, the underlying mechanism remains unknown (Liu H. et al., 2022). Studies have shown that the stiffness of Aβ plaque-associated tissues is increased, and the mechanosensitive ion channel Piezo1 is selectively upregulated in Aβ plaque-associated microglia (Hu et al., 2023). Piezo1 further sensory stiffness stimulates Aβ fibrils, which subsequently induces Ca2+ influx and promotes microglia aggregation, phagocytosis, and Aβ plaque compaction (Hu et al., 2023). The reduction in Piezo1 expression in microglia or the impairment of their ability to detect stiffness may play a decisive role in the progression of plaque pathology (Ikiz et al., 2024). Given the continued accumulation of Aβ plaques with age and disease progression, it is also suggested that microglial clearance of misfolded protein aggregates gradually fails over time (Blaschke et al., 2020). Piezo1-deficient microglia lead to worsening Aβ pathology and cognitive decline (Hu et al., 2023). Changes in Piezo1 expression in both astrocytes and microglia have been confirmed in AD animal models and patients with AD. Although the importance of these results should not be overlooked, it should be emphasized that these effects were examined in a microglial cell line and need to be further validated in whole animals. How these changes are related to the specific pattern of Piezo1 activity remains unclear (Ivkovic et al., 2022; Liu H. et al., 2022). Further studies are still needed in the future to determine the factors that affect the clearance of microglia and Aβ plaques.

According to the aforementioned description of the molecular mechanism, in AD, Aβ plaques can be regulated by regulating the expression of Piezo1 channels in the central nervous system (CNS). The activity of microglia can be modulated by designing specific agonists or inhibitors targeting Piezo1, thereby enhancing their ability to clear Aβ plaques. Gene therapy can also be used with gene-editing technology (such as CRISPR/Cas9) in order to regulate the expression of Piezo1, especially in the early stages of AD. It can upregulate Piezo1 to enhance the function of microglia, which is expected to slow or reverse cognitive decline. Another option is mechanical stimulation therapy, based on the sensitivity of the Piezo1 channel to mechanical stimulation, specific physical therapy or mechanical stimulation can be developed to regulate the activity of microglia by altering the mechanical properties of the microenvironment in the CNS, thereby promoting the clearance of Aβ plaques.

### 4.2 Pathological expression of Piezo1 in glioma

Gliomas are the most common primary malignant tumors of the CNS in adults (Chen et al., 2018; Louis et al., 2016; Qu et al., 2020b; Zhou et al., 2020). The annual incidence of Gliomas account for approximately 78.3% of all malignant tumors (Sung et al., 2021). Gliomas are classified as low-to high-grade (grades I–IV) based on disease progression (Chen et al., 2018; Zhou et al., 2020), with a higher grade indicating a more serious condition. Grade IV glioblastoma (GBM) is the most frequently occurring aggressive primary brain tumor in adults (Umesh et al., 2014), and it has the highest annual incidence rate, accounting for 48.6% of all primary malignant central system tumors (Sung et al., 2021). Traditional treatments including chemotherapy, surgery, and irradiation, are ineffective for GBM, and the median survival time of patients with gliomas is no longer than 14 months (Chen et al., 2018; Zhou et al., 2020). As GBM has a low cure rate and imposes a heavy socioeconomic burden, it is important to identify the underlying pathogenic mechanisms.

**Figure 2:**
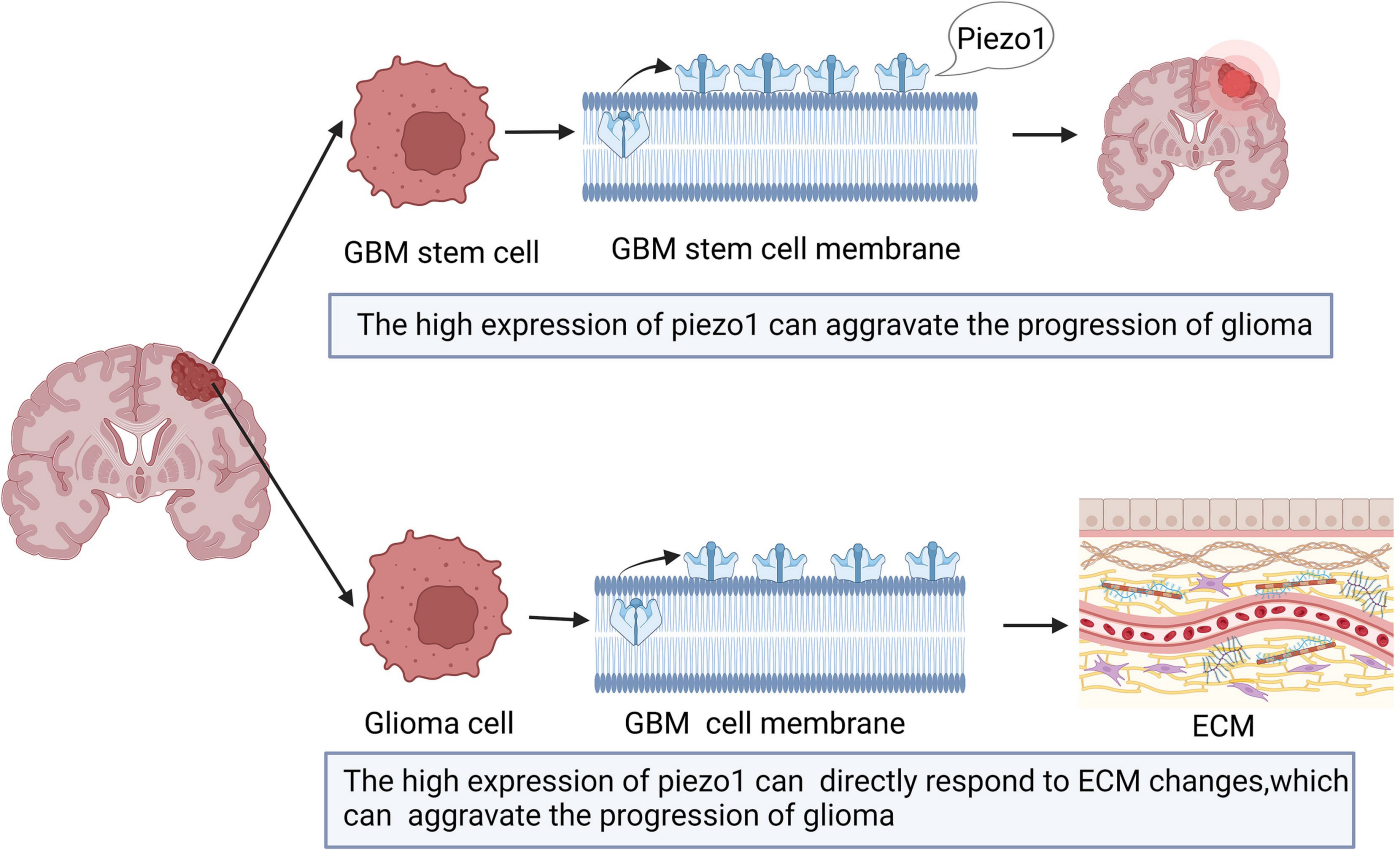
Schematic diagram of the action of Piezo1 on Glioma. The expression of Piezo1 is up-regulated in GBM stem cells, which is caused by the stiffer mechanical environment. And the higher the Piezo1 expression, the more the ECM environment will change. These changes can also aggravate the progression of glioma.

The expression of Piezo1 is increased in multiple malignant diseases (Jiang et al., 2017; Li et al., 2015). As shown in Figure 2, previous studies have shown that Piezo1 is highly expressed in glioma, and its expression is negatively correlated with patient survival (Chen et al., 2018; Qu et al., 2020b; Zhou et al., 2020). Piezo1 is one of a major ion channel responsible for the mechanical sensitivity of GBM stem cells (Chen et al., 2018). Glioma cells rely on Piezo1 for mechanical sensing. A stiff mechanical environment upregulates Piezo1 expression to further elevate tumor tissue mechanosensation and aggravate glioma progression (Chen et al., 2018). The expression of Piezo1 is significantly higher in GBM than in lower-grade gliomas (WHO grades II and III) (Zhou et al., 2020). This difference can be explained by the fact that GBM has a stiff mechanical environment in the brain, which upregulates the expression of Piezo1 (Chen et al., 2018).

The expression of Piezo1 can result in mechanical stimulation between glioma cells and promote the occurrence and development of tumors (Chen et al., 2018). Enhanced stiffness between glioma cells and the tumor microenvironment regulates Piezo1 activation and promotes pathological processes (Hong et al., 2023). In addition, the matrix metalloproteinase, tissue inhibitor of metalloproteinase, mitogen-activated protein kinase, and phosphocarnosin 3 kinase families are positively correlated with the high expression of Piezo1 in the pathological process (Zhou et al., 2020).

Piezo1 is also associated with the tumor microenvironment (Zhou et al., 2020). Mechanical stimulation, sensed by tumor and neighboring cells, is also provided by the tumor microenvironment and influences aspects of tumor progression and metastasis for unknown reasons (Hanahan and Coussens, 2012; Quail and Joyce, 2013). The expression of Piezo1 is highly correlated with tumor microenvironment-related genes that encode proteins involved in ECM organization, angiogenesis, and cell migration (Zhou et al., 2020). Thus, higher Piezo1 expression levels indicate subtle changes in the ECM environment. Therefore, tumor cells can directly respond to ECM changes caused by high expression of Piezo1 (Zhou et al., 2020; Figure 2).

The expression of Piezo1 is positively linked to gliomas, and inhibiting the expression of Piezo1 with drugs may be a treatment strategy for gliomas (Zhou et al., 2020). Past studies have shown that Ruthenium red, Gd^3+^, and Grammostola spatulata spider toxin (GsMTx4) may inhibit the expression of Piezo1 and reduce cell death in response to mechanical injury (Zhong et al., 2018; Zhou et al., 2020). However, GsMTx4 is a nonspecific inhibitor that may have unwanted effects on its expression in normal human tissues. Therefore, future studies with larger sample sizes are needed for the transition to preclinical studies to better elucidate the potential role of this inhibitor.

Based on the molecular mechanism of Piezo1, it is possible to clinically consider blocking the mechanosensitivity of tumor cells by inhibiting the activity of Piezo1 channels, thereby slowing tumor progression. Studies have shown that compounds such as ruthenium red, Gd3+, and the spider toxin from Grammostola spatulata (GsMTx4) may effectively inhibiting the expression of Piezo1 and reducing mechanical damage-induced cell death. These findings lay the foundation for the development of Piezo1 target-based therapeutics for gliomas. Although studies have demonstrated the potential of Piezo1 inhibitors under laboratory conditions, translating these results to clinical applications faces multiple challenges. First, current inhibitors such as GsMTx4 are nonspecific inhibitors that may adversely affect Piezo1 expression in normal tissues, increasing the risk of treatment side effects. Therefore, more selective and specific Piezo1 inhibitors need to be developed to reduce their effects on non-target tissues. Second, the regulation of Piezo1 expression by the mechanical environment and its mechanism of action in the tumor microenvironment are not fully understood. The mechanical stiffness of glioma tissues and other factors in the tumor microenvironment, such as extracellular matrix (ECM) remodeling, angiogenesis, and cell migration, may promote tumor progression through Piezo1 activation. Therefore, an in-depth understanding of these interactions is essential for developing effective therapeutic strategies. However, drug delivery remains challenging. Effective delivery of Piezo1 inhibitors to tumor regions in the brain and ensuring drug stability and efficacy remain challenges to be addressed in clinical translational research.

The mechanism of regulation of Piezo1 expression by the tumor microenvironment, especially the extracellular matrix and angiogenic process, can be further investigated by exploring the interaction of Piezo1 with the tumor microenvironment. This will help to understand the complexity of tumor progression and provide a basis for multi-targeted therapeutic strategies. Optimization of drug delivery systems could also be used to effectively deliver Piezo1 inhibitors to CNS tumor sites through nanotechnology or other advanced drug delivery systems, thereby improving the precision and effectiveness of treatment.

### 4.3 Pathological expression of Piezo1 in neuroinflammation

Neuroinflammation in the CNS is characterized by a variety of pathological changes such as elevated levels of proinflammatory cytokines, peripheral leukocyte infiltration, and nerve tissue damage (Estes and McAllister, 2014; O'Callaghan et al., 2008). As previously described, astrocytes and microglia in the brain are involved in neuroinflammation.

**Figure 3:**
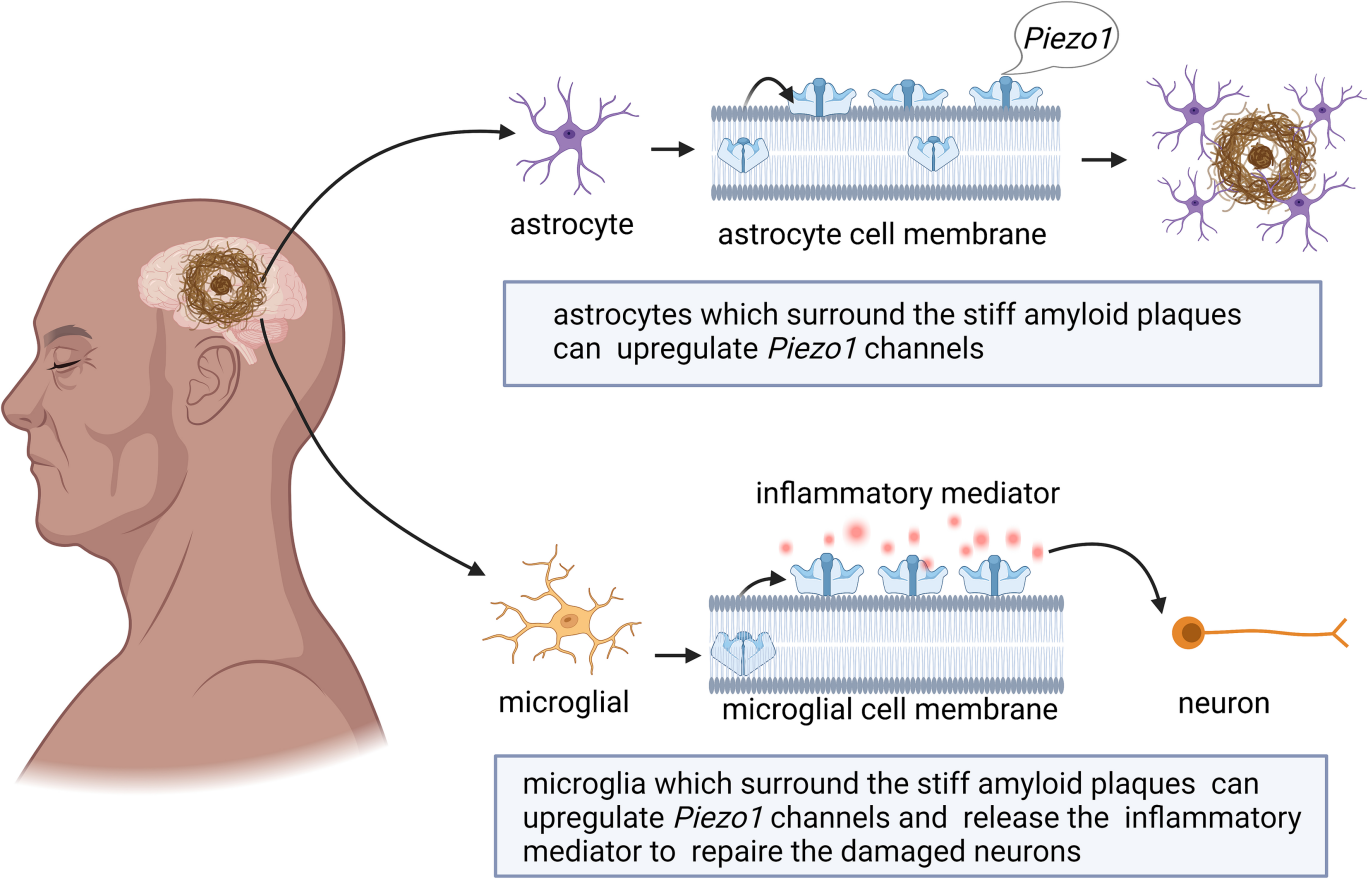
Schematic diagram of the action of Piezo1 on neuroinflammation in the brain. The expression of Piezo1 is up-regulated in astrocytes under the environment of LPS. Piezo1 channels will increase the oscillations of intracellular Ca^2+^ and inhibit the release of proinflammatory cytokines. The up-regulated expression of Piezo1 will also reduce the migration rate of astrocytes in an inflammatory state.

Astrocytes change in the mechanical properties of the surrounding microenvironment during or after CNS disease (Bowers et al., 2019). Piezo1 expression increases in astrocytes in response to increased nerve inflammation (Velasco-Estevez et al., 2020). For example, intraperitoneal injection of lipopolysaccharide (LPS) induces proinflammatory factor expression, Aβ accumulation, and increased cognitive deficits (Zhao et al., 2019). However, the high expression of Piezo1 induced by LPS may inhibit the release of inflammatory cytokines (e.g., TNF*α*, IL-1β, and CX3CL1) in the CNS. Piezo1 expression in cortical astrocytes is induced by LPS (Velasco-Estevez et al., 2020), and LPS exposure can upregulate Piezo1 expression in a modest proportion of astrocytes (approximately 25%) (Velasco-Estevez et al., 2020). As shown in Figure 3, activation of Piezo1 channels increases the oscillations of intracellular Ca^2+^ and inhibits the release of proinflammatory cytokines (Velasco-Estevez et al., 2020). Therefore, Piezo1 in astrocytes may play a role in inhibiting CNS inflammation.

As shown in Figure 3, high Piezo1 expression in astrocytes reduces the migration rate of astrocytes in the inflammatory state (Velasco-Estevez et al., 2020). Astrocytes do not normally migrate in healthy adult brains (Zhan et al., 2017), however, they can change shape in neuroinflammatory states and become hypertrophic, contributing to glial scarring (Buffo et al., 2010). Migration of LPS-stimulated astrocytes is slowed upon exposure to the Piezo1 activator Yoda1. Therefore, activation of Piezo1 channels may reduce the transition of astrocytes from an anti-inflammatory state to a proinflammatory state, thereby suppressing inflammation.

Unlike astrocytes, microglia are highly mobile under both physiological and pathological conditions and are capable of sensing mechanical signals in the microenvironment (Ayata and Schaefer, 2020; Bruno et al., 2021; Malko et al., 2022). Microglial cells in the CNS play dual roles in supporting neuronal cell activity and mediating neuroinflammation (Drummond et al., 2017; Malko et al., 2022). The activation of Piezo1 inhibits LPS-induced microglial cell activation and proinflammatory mediator (TNF-α and IL-6) production (Velasco-Estevez et al., 2018; Figure 3).

Although the mechanism of action is still unclear, Piezo1 channel activation in both microglia and astrocytes inhibits proinflammatory phenotypes, including the production of proinflammatory cytokines. We hope that future studies will help elucidate this mechanism.

These results suggest that Piezo1 is highly expressed in neuroinflammation and that Piezo1 channels may be a potential target for the treatment of neuroinflammation. Future studies should investigate the potential anti-inflammatory mechanism of Piezo1 in astrocytes in more detail to determine whether Piezo1 channels are novel therapeutic targets for the treatment of neuroinflammatory diseases in the brain.

The aforementioned study demonstrated that Piezo1 channels are highly expressed during neuroinflammation in the central nervous system (CNS) and inhibit the release of pro-inflammatory cytokines. These findings suggest that Piezo1 is a potential target for treating neuroinflammation. Clinically, drugs targeting Piezo1, such as Piezo1 agonists (e.g., Yoda1), can be used to reduce neuroinflammation, thereby protecting neurons from neurodegenerative diseases. However, in the treatment of neuroinflammation, single-target interventions may not completely control the complex inflammatory responses. Therefore, combining Piezo1 agonists with other anti-inflammatory drugs should be explored in the future with the aim of generating a synergistic effect in order to simultaneously inhibit the inflammatory response from multiple pathways and improve therapeutic efficacy.

### 4.4 Pathological expression of Piezo1 in ischemic stroke

Stroke is the third most common disease worldwide and one of the leading causes of disability in adults (Guo et al., 2021; Krivoshein et al., 2022; Yang et al., 2021). Ischemic strokes account for 75–85% of all strokes (Xu et al., 2020). Ischemic stroke is caused by the blockage of a blood vessel that interrupts blood flow to the brain and subsequently affects its downstream branches, starving the brain tissue of glucose and oxygen, causing cerebral edema, and headache (Hansen et al., 2015; IHS, 2018; Harriott et al., 2020; “Headache Classification Committee of the International Headache Society (IHS) The International Classification of Headache Disorders, 3rd edition,” 2018). Headaches after stroke significantly impact recovery, quality of life, and survival rates (Balami et al., 2011). Headaches after stroke may cause severe nerve cell death and further harm the patient’s health.

Cerebral edema caused by ischemic stroke exerts mechanical forces on the surrounding brain tissue (Guo et al., 2021; Krivoshein et al., 2022). Piezo1 channels convert the changes in these mechanical forces into electrical or chemical signals (Guo et al., 2021). The upregulated expression of Piezo1 is associated with edema (Krivoshein et al., 2022). Reducing the expression of Piezo1 prevented artery clots in rats and reduces the size of infarcts, which may represent a treatment approach for ischemic stroke (Krivoshein et al., 2022).

**Figure 4:**
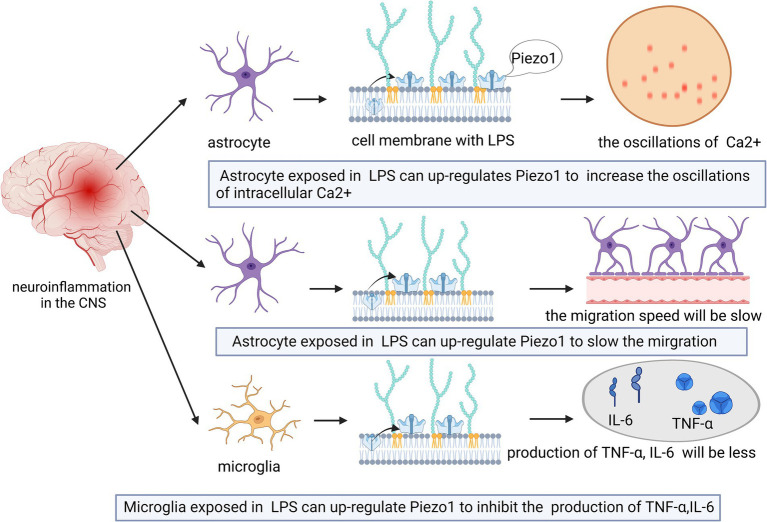
Schematic diagram of the action of Piezo1 on ischemic stroke in the CNS. Cerebral edema caused by ischemic stroke can lead to the up-regulation of Piezo1 expression. The degree of up-regulation is related to the size of edema. The more severe the brain edema, the stronger the expression of Piezo1.

Past studies have shown that hypoxia-inducible factor 1-alpha (HIF1*α*) is an essential protein in Piezo1/calcium signaling, ferritin pendulous regulation, and cerebral ischemia (Guo et al., 2021; Yang et al., 2018), and HIF1α expression is increased in neurons and astrocytes after cerebral ischemia (Hirayama and Koizumi, 2017). The upregulation of Piezo1 may trigger the expression of HIF1α and lead to increased uptake of iron by the transferrin receptor (TFR), which may further aggravate ischemic stroke (Guo et al., 2021; Figure 4). However, this hypothesis has not yet been proven, and future studies are required in order to test it.

Magnetic nanobubbles (MNBs) have been used as controllable mechanical probes to tag NSCs. When internalized by NSCs, MNBs activate Piezo1 channels, leading to Ca^2+^ influx, which activates the expression of BMP2 and increases the phosphorylation of SMAD, adjusting the expression of differentiation genes to prevent NSCs from expressing genes that promote cancer (Li J. et al., 2022).

Taken together, these studies demonstrated that high Piezo1 expression is unfavorable for the pathology of ischemic stroke. Therefore, the modulation of Piezo1 expression in ischemic stroke may be a therapeutic strategy. Further research focusing on this aspect is required.

A summary of the relationship between the expression of Piezo1 and the aforementioned four nervous system diseases is shown in Table 1.

**Table 1:** The expression of Piezo1 is linked to four CNS diseases.

Diseases	Study	Target cells	Subject of experiment	Variatin of Piezo1	Mechanical factors	Results	Related mechanisms
AD	(2, 4)	Astrocytes	Animal models and patients	Upregulate	Stiff amyloid plaques	Astrocytes proliferate to engulf the plaques	Unknown reasons
AD	(1, 3)	Microglia	Animal models and patients	Upregulate	Stiff amyloid plaques	Affect the immune activity of microglia to release more inflammatory mediators	Unknown reasons
Glioma	(6, 8)	GBM stem cells	Animal models and patients	Upregulate	Stiffer mechanical environment	Elevate tumor tissue mechanosensation to aggravate glioma progression	The stiffer mechanical environment upregulates Piezo1 expression
Glioma	8	Glioma cells	Animal models and patients	Upregulate	The change of ECM environment	Proliferation of tumor cells caused by ECM changes	The high expression of piezo1 caused ECM environment changes
Neuroinflammation	14	Astrocytes	Animal models and patients	Upregulate	LPS	Inhibit the release of proinflammatory cytokines	Increase the oscillations of intracellular Ca2+
Neuroinflammation	16	Astrocytes	Animal models and patients	Upregulate	Yoda1/LPS	Inhibit the migration of astrocytes into neuroinflammatory cells	Inhibit the migration of astrocytes to suppress inflammation
Ischemic stroke	(18, 20)	Brain tissue cells	Animal models and patients	Upregulate	Mechanical forces	Increase the size of infarcts	Piezo1 can converts changes in the force into an electrical or chemical signal
Ischemic stroke	20	brain tissue cells	Animal models and patients	Upregulate	Mechanical forces	Trigger the expression of HIF1α to aggravate the condition of ischemic stroke	Waiting for verification
Ischemic stroke	22	NSCs	Animal models and patients	Upregulate	Internalization of NSCs	Prevent NSCs from expressing in the direction of cancer	Piezo1 can activate the expression of BMP2, resulting in increased phosphorylation of Smad

## 5 Piezo1 as a potential therapeutic target for CNS diseases

As previously described, the overactivation of Piezo1 is highly correlated with the onset and progression of many CNS disorders. Therefore, efficient inhibitors or antagonists should inhibit Piezo1 channel activity by preventing its overexpression, which provides a clear method for the treatment of Piezo1-associated diseases (Coste et al., 2010; Lee et al., 2021; Velasco-Estevez et al., 2020).

### 5.1 The related antagonists of Piezo1

**Figure 5:**
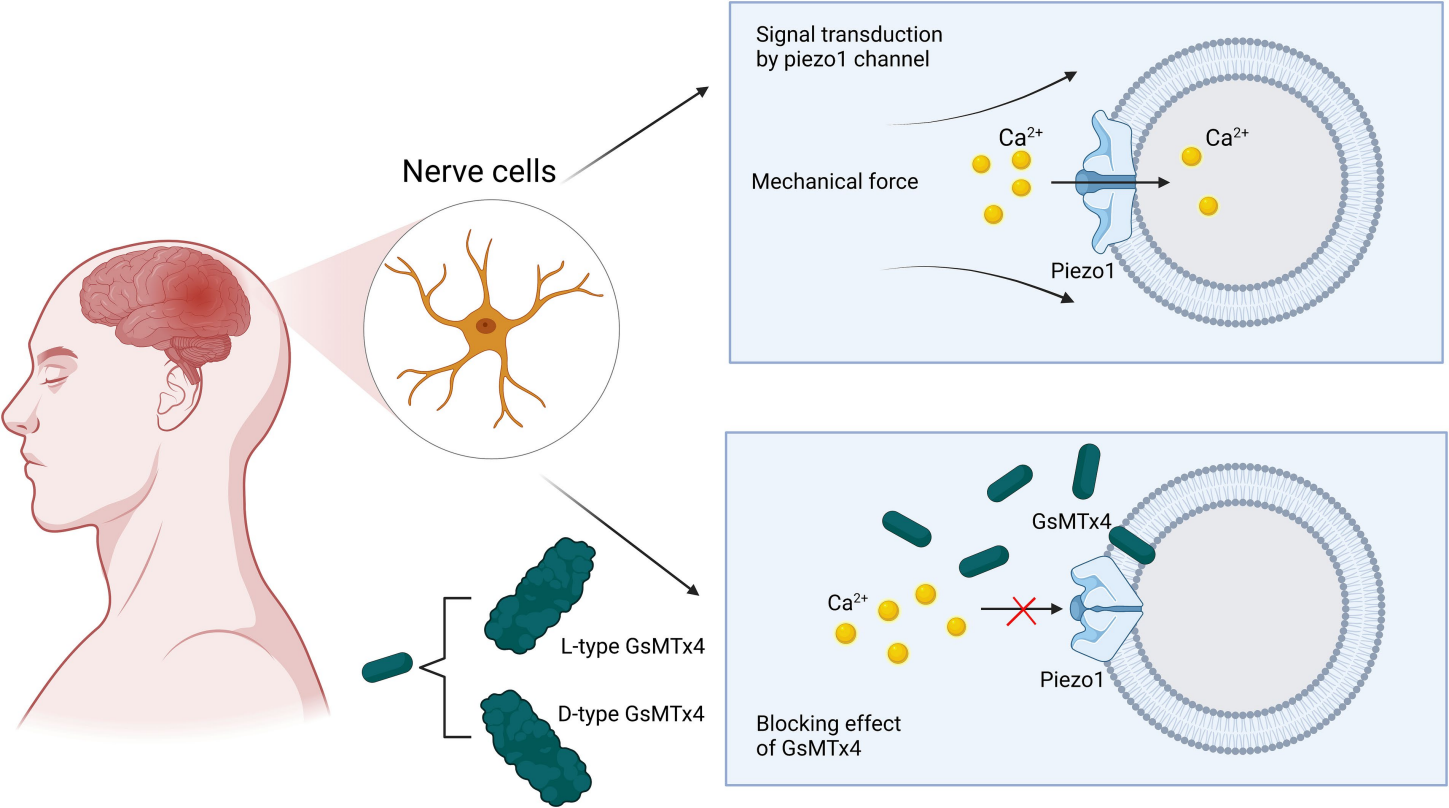
Schematic representation of GsMTx4 inhibition of Piezo1 channels. Under normal physiological conditions, mechanical pressure applied to the cell membrane can cause Piezo1 channels to respond and open rapidly to participate in mechanical signal transduction. When the antagonist GsMTx4 is involved in signal transduction in these normal cells, it can cause the closure of Piezo1 channels, preventing calcium ions from entering the neuronal cells and interrupting signal transduction. The mechanism by which this process occurs is the proximity and insertion of GsMTx4 into the phospholipid bilayer in the vicinity of the channel, causing a change in membrane tension and ultimately inducing the closure of the channel.

Originally isolated from the venom of tarantulas, GsMTx4 is an amphipathic peptide with six cysteine residues and is the only known antagonist that specifically targets cationic mechanosensitive channels (MSCs) (Bosmans and Swartz, 2010; Lee and MacKinnon, 2004; Suchyna, 2017). GsMTx4 possesses a conserved inhibitory cysteine junction (ICK) backbone, which, as for all ICK peptides, has both hydrophobic and hydrophilic surfaces that facilitate their adsorption to lipid bilayers and direct binding to gating elements located on the membrane to alter channel dynamics (Bosmans and Swartz, 2010; Kimura, 2021; Posokhov et al., 2007). Many other ICK peptides have been isolated from tarantula venom. However, GsMTx4 has been shown to have a higher positive charge (+5) and a relatively higher hydrophobicity and lysine content. Its inhibitory effect was not stereospecific, and the inhibitory effects of the L- and D-types were roughly equal. Therefore, GsMTx4 stands out from the other ICK peptides (Caputo and London, 2003; Copp et al., 2016; Lee and MacKinnon, 2004; Ostrow et al., 2003). The bilayer tension of lipid membranes regulates their density and thickness to play an activating or inhibiting role in MSCs (Cox et al., 2016; Markin and Sachs, 2015; Phillips et al., 2009). The GsMTx4 peptide does not function by binding to gating elements on the membrane, but causes local changes in biofilm properties in the vicinity of the channel, which leads to MSC inhibition (Lee and MacKinnon, 2004). As shown in Figure 5, GsMTx4 approaches the piezoelectric 1 track and inserts itself into the lipid bilayer, which reduces membrane tension near the track and exerts a nonspecific inhibitory effect (Gnanasambandam et al., 2017). The use of GsMTx4 before mechanical stimulation of the Piezo1 ion channel produced almost 100% inhibition, which suggested that the change in membrane properties near the channel was accompanied by the closure of the Piezo1 channel (Bae et al., 2011). A recent preclinical study showed that the administration of GsMTx4 effectively inhibited Piezo1 overexpression, regulated mechanosensitive Ca^2+^ channels, and prevented CNS demyelination (Velasco-Estevez et al., 2020). One study exploring the correlation between Piezo1 and inflammatory responses found that blockade of Piezo1 with GsMTx4 led to increased expression of cytokines such as TNF-α, Il-1*β*, and Il-6, and alleviation of CNS inflammatory effects, which also revealed the role of GsMTx4 in neuroinflammation (Shinge et al., 2022). Massive accumulation of β-amyloid plaques is often seen in the brains of patients with AD, which induces increased expression of Piezo1. This increased expression disrupts the vascular endothelium and substantially increases the permeability of the blood–brain barrier, ultimately leading to the exacerbation of AD (Velasco-Estevez et al., 2022; Velasco-Estevez et al., 2018). High Piezo1 expression causes glioma cells to spread at a faster rate. Therefore, reducing Piezo1 gene using GsMTx4 can effectively mitigate the invasive effects of glioma cells on surrounding normal cells (Chen et al., 2018). Additionally, in a study of *in vitro* and *in vivo* stroke models, the use of the Piezo1 agonist Yoda1 led to increased cell death, whereas the use of the Piezo1 antagonist GsMTx4 led to decreased cell death following hypoxia. Inhibition of Piezo1 by GsMTx4 in the cerebral cortex of rats after ischemia/reperfusion attenuates cell viability inhibition and apoptosis, increases intracellular calcium levels, and enhances calpain activity (Bryniarska-Kubiak et al., 2023). One question is why GsMTx4 affects many key physiological processes *in vitro*, but has little effect on normal physiological processes *in vivo*, yet has a strong therapeutic effect on pathology. Chronic GsMTx4 exposure restricts the entry of GsMTx4 into the mechanical domains containing mesenchymal stem cells (MSCs) in undisturbed membranes. GsMTx diffuses to its target by binding to the bulk film, a process that may require a critical concentration to buffer the local tension. Under pathological conditions, as well as in non-physiological *in vitro* experiments, the membrane diffusion barrier may be breached. The time required for GsMTx4 to inhibit the whole-cell piezoelectric current suppression is relatively long. The membrane barrier may restrict the entry of GsMTx4 into MSCs except under pathological conditions, which could explain the lack of side effects under normal physiological stress (Suchyna, 2017).

### 5.2 The related nonspecific blockers of Piezo1

Metallic gadolinium, and ruthenium red reagents are also nonspecific blockers of Piezo1 channels (Li X. et al., 2022). As a trivalent lanthanide, gadolinium inhibits a variety of MSCs, including Piezo1 channels, TREK-1, and voltage-gated sodium channels (Biagi and Enyeart, 1990; Coste et al., 2010; Maingret et al., 2000). Notably, the mechanism of action is currently unknown, and its concentration may be a major factor in its inhibitory effect (Hamill and McBride, 1996). Recent studies have noted the existence of specific antagonists of Piezo1 such as Tubeimoside1 and Dooku1 (Shinge et al., 2022). Piezo1 is also selectively activated by the small molecule Yoda1; however, its activation mechanism is unknown (Botello-Smith et al., 2019). While Tubeimoside1 can inhibit the activation of Piezo1 channels by endogenous Yoda1 when it acts alone, it does not affect the activity of Piezo1 channels, suggesting that the action of Tubeimoside1 is dependent on the presence of Yoda1, which has also been observed in Dooku1 (Evans et al., 2018; Liu S. et al., 2020). The mechanism of action is not the same for both agents. Dooku1 inhibits the Piezo1 channel by modifying the pyrazine ring of Yoda1 (Evans et al., 2018). The inhibition rate of Tubeimoside1 showed a decreasing trend with increasing Yoda1 concentration, which suggests that it competes with Yoda1 for the binding site to achieve inhibition (Liu S. et al., 2020).

### 5.3 Piezo1 agonists

At present, there are only a few kinds of piezo1 agonists. Yoda1was the first chemical activator developed to promote the open conformation of Piezo1. Yoda1 binds allosterically to A pocket about 40 A from the central pore and acts as a molecular wedge to facilitate force-induced conformational changes by lowering the mechanical activation threshold of the channel (Botello-Smith et al., 2019). Yoda2 is a structurally modified version of Yoda1 that significantly enhances Piezo1 agonist activity at concentrations similar to those of Yoda1 (Endesh et al., 2023). Similar to Yoda1, Yoda2 stabilizes the open conformation of Piezo1 with a negative charge at physiological pH and a more desirable pharmacokinetic profile (Parsonage et al., 2023).

In addition to Yoda1 and Yoda2, Piezo1 is activated by Jedi1 and Jedi2 (Botello-Smith et al., 2019), but Jedi1 and Jedi2 cannot cross the cell membrane; Therefore, Piezo1 can only be activated outside the cell (Ikiz et al., 2024). Yaddle1 is a recently discovered agonist that may lower the mechanical activation threshold of the channel and stabilize its open conformation like Yoda1/2 agonists (Goon et al., 2024).

Until now, the clinical application of piezo1 related drugs has not been perfect, and the related biological experiments are also relatively limited. Further experimental research is required in order to promote this process.

## 6 Conclusion and prospects

Current studies suggest that the Piezo1 channel is a genetic product closely associated with intercellular mechanistic responses that contribute to the development and various physiological activities of the CNS as well as the development of various disease states. Piezo1 regulates the number of neuroepithelial cells in their physiological state and maintains their integrity. Piezo1 has a dual influence on axonal growth in the brain, underlying the limited repair capacity of sensory and motor neurons (Koser et al., 2016; Zhou et al., 2020). This prevents uncontrolled growth of neurons and reduces energy loss. They also play a role in the perception of the mechanical properties of the microenvironment of neural progenitor cells. Piezo1 is expressed in the dorsal ganglion of mice and likely primarily mediates nociception (Roh et al., 2020; Wang et al., 2019). Piezo1 mediates Ca^2+^ transients to establish vascular networks in the brain and plays an important role in the mechanosensing of capillary blood flow (Cudmore and Santana, 2022; Liu T. T. et al., 2020). However, when it is absent, as in Piezo1 KO embryos, the level of cholesterol synthesis in the CNS and the levels of neutral lipids and cholesterol in NSCs are reduced (Nourse et al., 2022). This reduction leads to a significant decrease in the number of neural precursor cells, immature neurons, mature neurons, and astrocytes, severe hippocampal volume defects, and a significant reduction in brain weight (Lee et al., 2021). When Piezo1 expression is upregulated, past experiments have shown that small injections of the Piezo1 channel agonist Yoda1 into the BF of mice produce the same pathological manifestations as those of sleep deprivation (Ma et al., 2022). High-dose injections of Yoda1 extend the meningeal arteries innervated by the trigeminal afferent nerve, which leads to the development of migraines (Dolgorukova et al., 2021).

In that, the expression of Piezo1 increases significantly with age, which is likely associated with aging-related neurological diseases. The stiffness of *β*-amyloid plaques in patients with AD produces mechanical stimulation of surrounding cells, and Piezo1 channels receive stimulation and activate astrocytes and microglia (Liu J. et al., 2021; Segel et al., 2019). However, there is positive feedback in regard to gliomas. Glioma clumps formed by GBM lead to the upregulation of Piezo1 expression, which, in turn, promotes the rapid proliferation and invasion of glioma cells, accelerates the spreading range and speed of the clumps, and creates a vicious cycle (Chen et al., 2018). Additionally, Piezo1 channels negatively regulate myelin formation in the CNS (Velasco-Estevez et al., 2020), and changes in mechanical forces may lead to the activation of Piezo1 channels, exacerbating ischemic brain injury (Guo et al., 2021). Piezo1 channels are also involved in tumor ECM organization, angiogenesis, and cell migration associated with the tumor microenvironment (Zhou et al., 2020).

Little is known about the signal transduction downstream of Piezo1. GsMTx4 is the only known antagonist that specifically targets cationic MSC inserted into the lipid bilayer, decreases membrane tension near the orbital, and exerts a nonspecific inhibitory effect. Metal gadolinium and ruthenium red are also Piezo1 blocking agents. However, their effects are non-specific (Li X. et al., 2022). Recent studies suggest that Tubeimoside1 is a specific antagonist of Piezo1. Tubeimoside1 is dependent on the presence of Yoda1, which was also observed with Dooku1, and may compete with Yoda1 for the binding site (Evans et al., 2018; Liu S. et al., 2020).

Many questions need to be addressed in order to gain a deeper understanding of the functions and signal transduction downstream of Piezo1. For example, how is Piezo1 involved in the nervous system development? The exact relationship between Piezo1-mediated mechanosensing and axonal growth in the brain remains unclear. How do stiff amyloid plaques in the brains of patients with AD and hard masses in gliomas stimulate the upregulation of Piezo1 expression in astrocytes and microglia, and how does Piezo1 overexpression proliferate in astrocytes and microglia? With the passing of time, rapid advances in high-throughput technologies, atomic force microscopy, membrane clamp electrophysiology, and cryo-electron microscopy, including other computational biology tools and gene editing techniques, will allow further studies on Piezo1 channels to answer these questions.

In conclusion, recent findings on the structural and functional relationships and genetic, physiological, and pharmacological properties of Piezo1 channels have largely contributed to our understanding of CNS mechanics. These findings will help to identify new therapeutic strategies for the treatment of several CNS disorders.

## Glossary

**Table:** 

Aβ	Beta-amyloid protein
ACC	Anterior cingulate cortex
AD	Alzheimer’s disease
AJs	Adhesion junctions
BF	Basal forebrain
CNS	Central nervous system
CSK	Cytoskeleton
DRG	Dorsal root ganglion
ECM	Extracellular matrix
EOAD	Early-onset Alzheimer’s disease
ETCs	Endothelial tip cells
FAZ	Focal adhesion zone
GBM	Glioblastoma
GsMTx4	Grammostola spatulata spider toxin
HCC	Hepatocellular carcinoma
ICK	Inhibitory cysteine junction
KO	Knockout
LOAD	Late-onset Alzheimer’s disease
LPS	Lipopolysaccharide
MA	Mechanically activated
MNBs	Magnetic nanobubbles
MSC	Mechanosensitive channel
NSCs	Neural stem cells
OPC	Oligodendrocytes
OPCs	Oligodendrocyte progenitor cells
PD	Parkinson’s disease
PECAM	Platelet endothelial cell adhesion molecule
SCI	Spinal cord injury
TFR	The transferrin receptor
